# Coexisting Situs Inversus Totalis and Immune Thrombocytopenic Purpura

**DOI:** 10.1155/2016/8605673

**Published:** 2016-02-14

**Authors:** Kemal Gundogdu, Fatih Altintoprak, Mustafa Yener Uzunoğlu, Enis Dikicier, İsmail Zengin, Orhan Yağmurkaya

**Affiliations:** ^1^Department of General Surgery, Sakarya University Research and Educational Hospital, Sakarya, Turkey; ^2^Department of General Surgery, Sakarya University Faculty of Medicine, Sakarya, Turkey

## Abstract

Situs inversus totalis is a rare congenital abnormality with mirror symmetry of mediastinal and abdominal organs. Immune thrombocytopenic purpura is an autoimmune disease with destruction of thrombocytes. This paper is presentation of surgical approach to a case with coexistence of these two conditions.

## 1. Introduction

Chronic immune thrombocytopenic purpura (ITP) is an autoimmune disease characterized by an immune response to thrombocyte membrane antigens. ITP is generally treated with surgery and steroids [1]. Situs inversus totalis is a rare congenital abnormality in which all of the mediastinal and abdominal organs are transposed to mirror symmetry of the normal anatomy. This paper reports the coexistence of these two rare conditions.

## 2. Case Presentation

**Figure 1:**
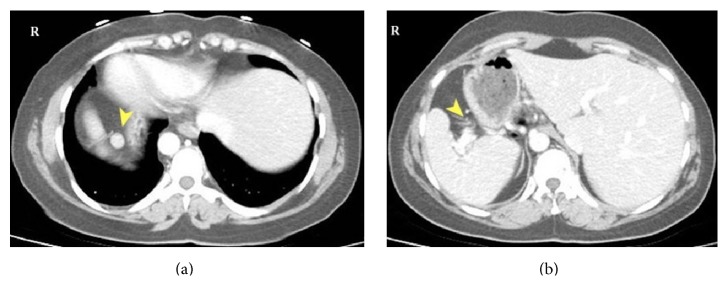
Abdomen CT; mirror view of intra-abdominal organs due to situs inversus, accessory spleen (arrow head (a)), and no pancreatic tissue in splenic hilum (arrow head (b)).

**Figure 2:**
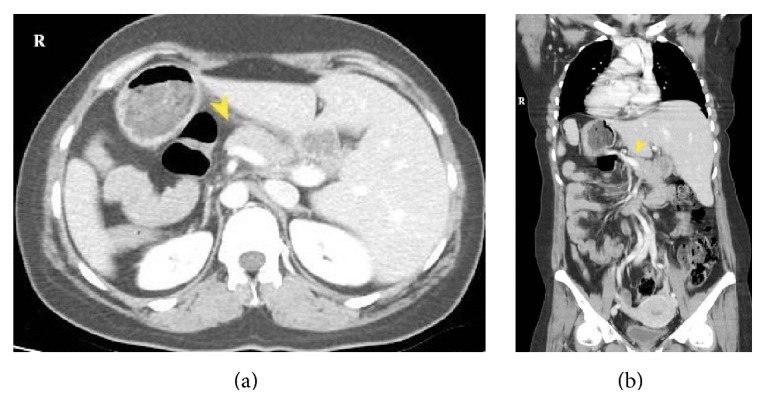
Abdomen CT; pancreatic tissue ends by bounds of superior mesenteric vein axis.

**Figure 3:**
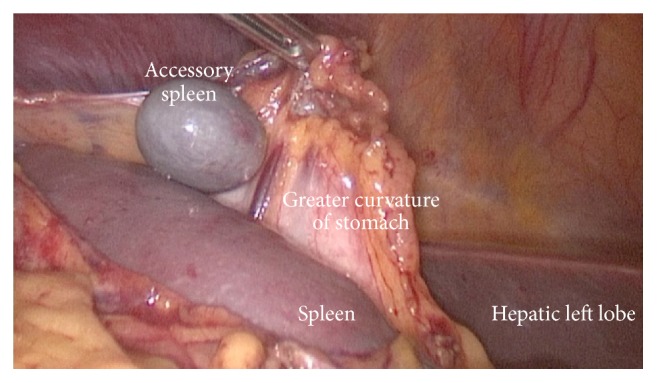
Intraoperative view; accessory spleen.

A 35-year-old woman was referred to our clinic with a diagnosis of ITP resistant to medical treatment in whom a splenectomy was indicated. For 7 years, she had been treated regularly with 1 mg/kg/day methylprednisolone and episodic intravenous immunoglobulin (IVIG) in the hematology clinic and had persistent thrombocytopenia of 3,000–60,000/mm^3^, ongoing menorrhagia, and ecchymosis. The patient had two healthy births after IVIG treatment. Preoperatively, dextrocardia was detected on an electrocardiogram and thoracoabdominal computed tomography (CT) showed situs inversus totalis. In addition to mirror imaging of the normal anatomic locations of all organs, the spleen measured 13 × 6 cm and there was a 1.5 cm accessory spleen in the right upper quadrant (Figure 1). Although no abnormalities were seen in the head and body of the pancreas, no pancreatic tail was seen. In other words, the pancreas had not crossed over to the right side of the superior mesenteric vascular axis and there was no pancreatic tissue near the splenic hilum (Figure 2). The patient underwent a laparoscopic splenectomy (Figure 3) with no complications or adverse events following surgery. She was discharged on the third postoperative day with thrombocyte count of 155,000/mm^3^.

## 3. Discussion

Immune (idiopathic) thrombocytopenic purpura is an autoimmune disease characterized by the destruction of thrombocytes or suppression of their production as a result of an immune reaction with thrombocyte membrane autoantigens [1]. Therapy is required when the thrombocyte count falls below 20,000/mm^3^ or if there are symptoms such as mucosal or vaginal hemorrhage when the count is in the range 20,000–50,000/mm^3^. In the event of nonresponse to medical therapy, the alternative therapy is splenectomy [2]. The treatment success rate after splenectomy ranges within 50–70%. Accessory spleens are found in 15% of the general population and should be detected in ITP patients when a splenectomy is planned to prevent incomplete removal of spleen tissue [3]. In our case, an accessory spleen was detected on preoperative CT and excised.

Situs inversus totalis is a rare condition with an incidence of 1/10,000 characterized by transposed organs and systems to mirror symmetry instead of the normal anatomy [4]. It is thought that an autosomal recessive genetic predisposition characterized as a defect on the long arm (q) of the 14th chromosome is responsible for organ transposition. Coexistence of SIT and various congenital abnormalities has been reported [5, 6]. The absence of a pancreatic tail in our case corresponded with the literature. In rare reported cases, patients with SIT undergo surgery for various indications. Anatomic congenital abnormalities result in surgeons practicing in unfamiliar territory, with undesirable outcomes if the surgeon is not aware of an abnormality, such as in emergency interventions. The pancreatic abnormality in our case did not cause any problems because it was identified preoperatively. Furthermore, the absence of pancreatic tissue near the splenic hilum facilitated isolation of the splenic vessels, eliminated the risk of pancreatic injury, and facilitated perioperative movements, contributing to an uneventful operation.

## 4. Conclusion

The coexistence of situs inversus totalis and ITP is very rare. We believe that a detailed radiological examination is very important for detecting accompanying anatomic abnormalities when surgical intervention is planned.
